# Supplementary material for: Small Cajal body-associated RNA 2 (scaRNA2) regulates DNA repair pathway choice by inhibiting DNA-PK
Source: Nat Commun. 2022 Feb 23;13:1015. doi: 10.1038/s41467-022-28646-5 (PMC8866460; doi:10.1038/s41467-022-28646-5)
Supplement: Supplementary file 1 — Supplementary Information [file 41467_2022_28646_MOESM1_ESM.pdf]

Supplementary figures and tables for

Small Cajal body-associated RNA 2 (scaRNA2) regulates DNA repair pathway choice by inhibiting DNA-PK

Sofie Bergstrand[1], Eleanor M. O'Brien[2,] Christos Coucoravas[2], Dominika Hrossova[2], Dimitra Peirasmaki[2], Sandro Schmidli[2], Soniya Dhanjal[2], Chiara Pederiva[2], Lee Siggens[1], Oliver Mortusewicz[3], Julienne J. O'Rourke[2] and Marianne Farnebo[1,2]*

This file includes

Supplementary Figs. 1-10

Supplementary Tables 1-4

Supplementary Fig. 1: Irradiation does not affect the total cellular level of scaRNA2

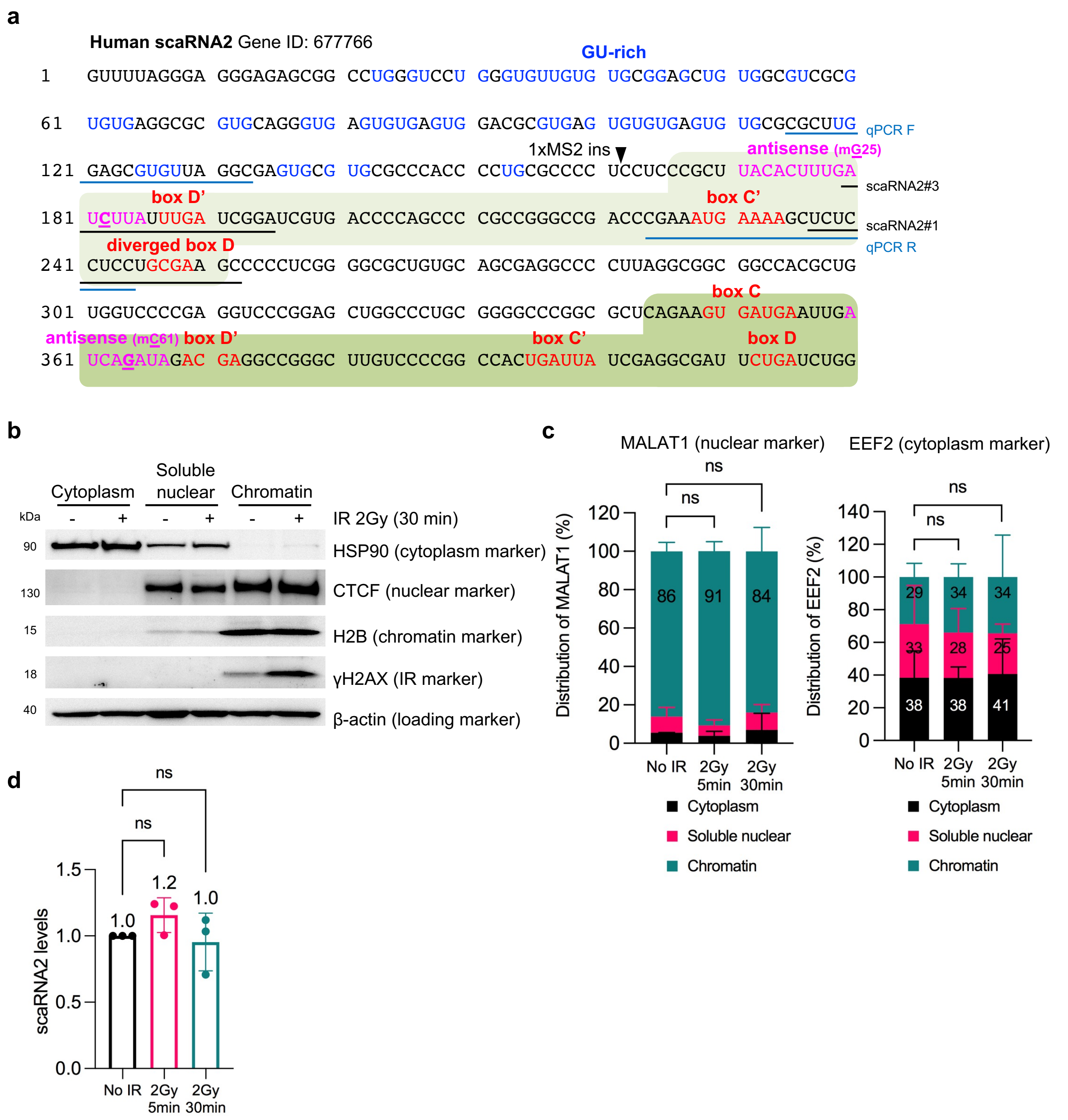

a, Sequence of human scaRNA2. The locations of UG/GU-rich dinucleotides are indicated in blue, the C, C′, D and D' boxes in red, predicted C/D domains [47] in light/dark green; and the U2 snRNA antisense regions in purple. The binding sites for qPCR primers are marked with blue lines, GapmeRs with black lines, and the site of MS2 loop insertion site by an arrow head. **b, c**, The purity of cytoplasmic, soluble nuclear and chromatin fractions prepared from untreated or irradiated (IR 2 Gy, 30 min recovery) U2OS cells was assessed by (b) western blotting or (c) qPCR of the marker proteins/RNAs indicated, (means ± SD, n=3), ns (not significant) as determined by one-way ANOVA and two-sided Dunnett's multiple comparisons

test. The large amounts of EEF2 mRNA recovered in the nuclear fraction may reflect the presence of endoplasmic reticulum (ER), which is continuous with the outer nuclear membrane and the primary site of general protein synthesis.

d, qPCR analysis of scaRNA2 expression in untreated or irradiated (2 Gy, 5 or 30 min recovery) U2OS cells. The levels of scaRNA2 were normalized to those of β-actin and are shown relative to the No IR sample (means ± SD, n=3), ns (not significant) as determined by one-way ANOVA and two-sided Dunnett's multiple comparisons test.

Source data are provided as a Source Data file.

Supplementary Fig. 2: Overexpressed scaRNA2-MS2 localizes to Cajal bodies and DNA breaks

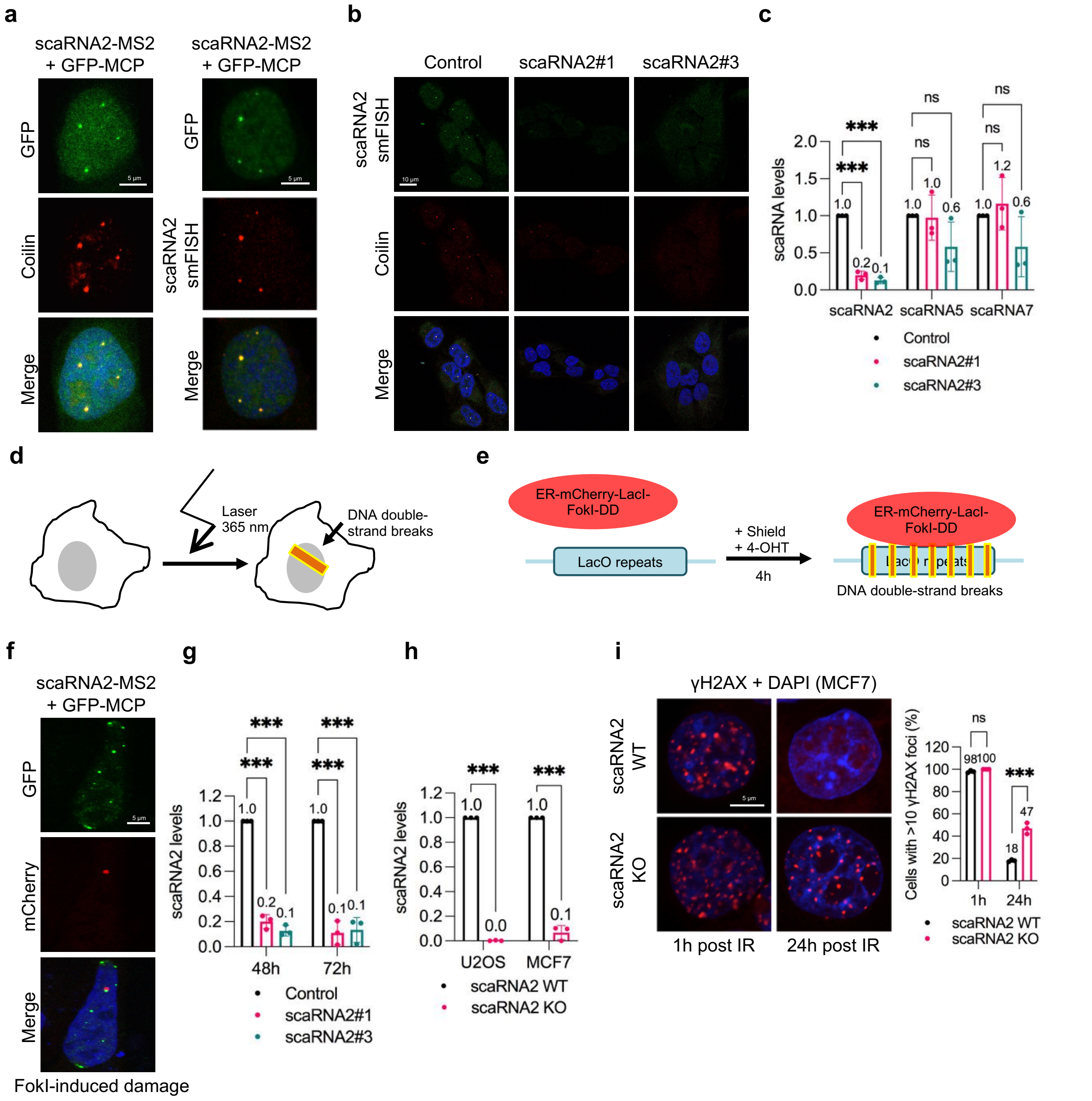

a, U2OS cells were co-transfected with plasmids containing GFP-MCP and MS2-tagged scaRNA2 for 24 h and then subjected to immunostaining for coilin or smFISH for scaRNA2 (n=3).

b, U2OS cells were transfected with GapmeRs for 48 h, fixed and subjected to immunostaining for coilin and smFISH for scaRNA2 (n=3).

c, U2OS cells were treated with the GapmeRs indicated for 48 h and then analyzed by qPCR for expression of scaRNA2, scaRNA5 and scaRNA7. The RNA levels were normalized to those of β-actin mRNA and are shown relative to the control sample (means ± SD, n=3), ***$p<0.001$,

ns (not significant) as determined by one-way ANOVA and two-sided Dunnett's multiple comparisons test.

d, Illustration of DNA double-strand breaks induced by laser micro-irradiation.

e, Illustration of the FokI endonuclease system in U2OS cells containing several hundred repeats of the Lac operator (LacO). The FokI protein is fused to mCherry, the Lac repressor (LacI), estrogen receptor (ER) and a destabilization domain (DD). Upon addition of Shield and 4-OHT, the fusion protein is stabilized and transported into the nucleus, where it binds to the LacO repeats and induces site-specific DNA double-strand breaks.

f, U2OS FokI cells were co-transfected with plasmids containing GFP-MCP and MS2-tagged scaRNA2 for 24 h and then treated with Shield and 4-OHT for an additional 4 h (n=3).

g, U2OS cells were treated with the GapmeRs indicated for 48 h or 72 h and then analyzed by qPCR for expression of scaRNA2. The RNA levels were normalized to those of β-actin mRNA and are shown relative to the Control sample (means ± SD, n=3), ***p<0.001 as determined by one-way ANOVA and two-sided Dunnett's multiple comparisons test.

h, qPCR analysis of scaRNA2 expression in wild-type (WT) or knock-out (KO) U2OS or MCF7 cells. The RNA levels were normalized to those of β-actin mRNA and are shown relative to the scaRNA2 WT sample (means ± SD, n=3), ***p<0.001 as determined by unpaired two-tailed t-test. Note, our U2OS WT/KO cells do not express Cas9, while the MCF7 WT/KO cells express this protein stably.

i, MCF7 scaRNA2 WT or KO cells were irradiated (2 Gy), fixed 1 or 24 h later, and subsequently immunostained for γH2AX. The graph below shows the percentage of 100-200 cells (means ± SD, n=3) whose nuclei contained >10 γH2AX foci, ***p<0.001, ns (not significant) as determined by unpaired two-tailed t-test.

Source data are provided as a Source Data file.

Supplementary Fig. 3: HR repair is restored by overexpression of scaRNA2 and loss of this RNA does not alter the cell cycle

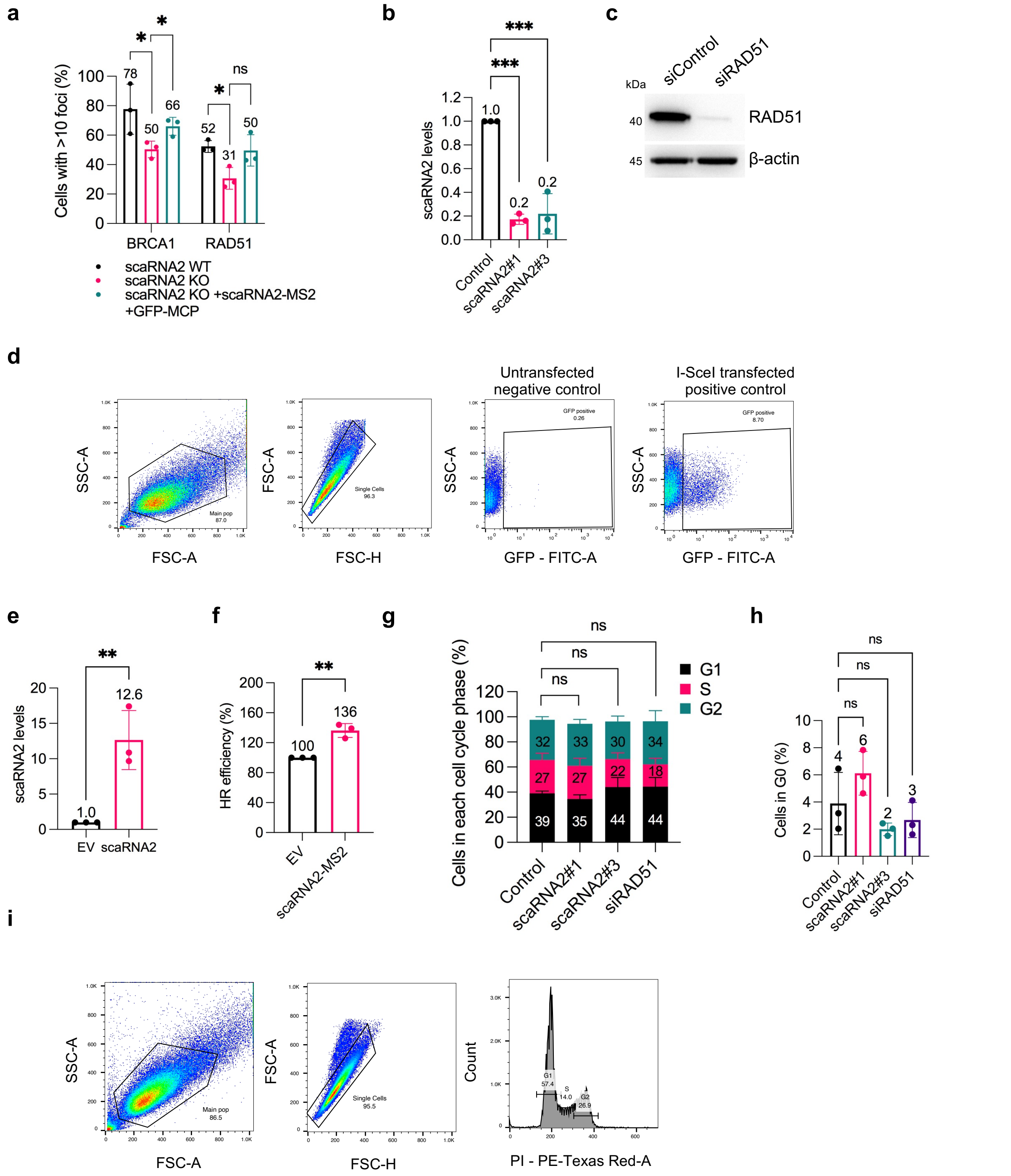

a, U2OS scaRNA2 WT or KO cells were either left untreated or transfected with GFP-MCP and MS2-scaRNA2 plasmids for 24 h, irradiated (2 Gy) , fixed 1 h later and immunostained for BRCA1 or RAD51. The graph shows the percentage of 100-200 untreated or GFP-positive cells (means ± SD, n=3) whose nuclei contained >10 IR-induced foci, *p<0.05, ns (not significant) as determined by one-way ANOVA and two-sided Dunnett's multiple comparisons test.

b, qPCR analysis of scaRNA2 expression in DR-GFP U2OS cells following knockdown for 48 h. The RNA levels were normalized to those of β-actin mRNA and are shown relative to the Control sample (means ± SD, n=3), ***p<0.001 as determined by one-way ANOVA and two-sided Dunnett's multiple comparisons test.

c, Western blotting of RAD51 and β-actin in DR-GFP U2OS cells following knockdown of RAD51 for 48 h. The blots shown are representative of 3 independent experiments.

d, Gating strategy for analysis of DR-GFP U2OS cells.

e, Expression of scaRNA2 in DR-GFP U2OS cells following transfection with the empty vector (EV) or scaRNA2 plasmid for 24 h, as determined by qPCR. RNA levels were normalized to those of β-actin mRNA and are shown relative to the EV sample (means ± SD, n=3), **p<0.01 as determined by unpaired two-tailed t-test.

f, The efficiency of HR following overexpression of MS2-scaRNA2 or an empty vector (EV) for 48 h. GFP expression was analyzed by flow cytometry. The values are shown relative to the EV sample (means ± SD, n=3), **p<0.01 as determined by unpaired two-tailed t-test.

g, Flow cytometric characterization of the cell cycle of DR-GFP U2OS cells treated with GapmeRs directed against scaRNA2 or siRNA against RAD51 for 48 h. The percentages of cells (means ± SD, n=3) in the G1, S and G2 phases of the cell cycle is shown, ns (not significant) as determined by one-way ANOVA and two-sided Dunnett's multiple comparisons test.

h, The percentage of DR-GFP U2OS cells (means ± SD, n=3) in the G0 phase following treatment with GapmeRs against scaRNA2 or siRNA against RAD51 for 48 h, as determined by flow cytometry, ns (not significant) as determined by one-way ANOVA and two-sided Dunnett's multiple comparisons test.

i, Gating strategy for cell cycle profiling.

Source data are provided as a Source Data file.

Supplementary Fig. 4: Loss of scaRNA2 does not alter expression of repair proteins

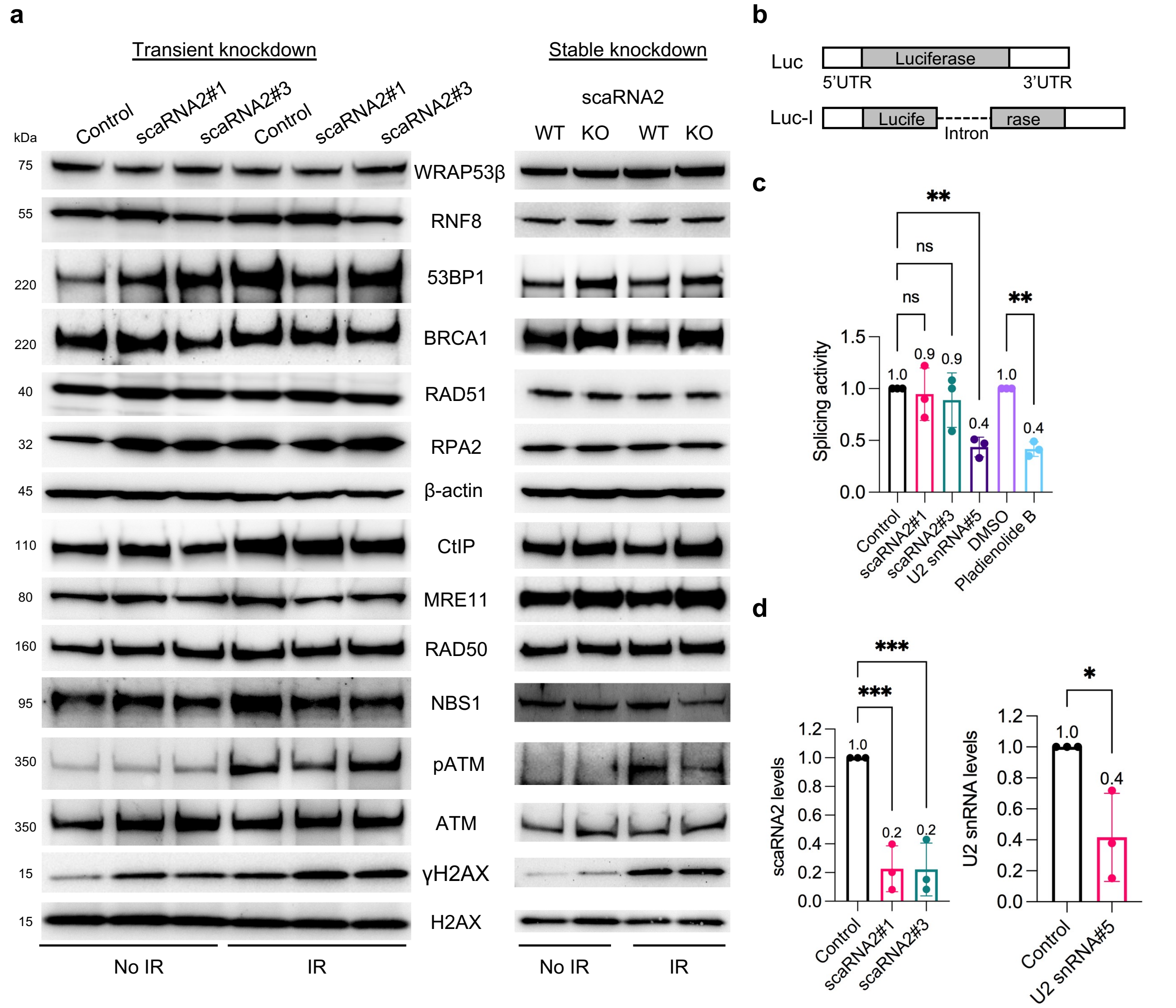

a, Western blotting of repair proteins in U2OS cells transiently or stably depleted of scaRNA2 with and without subsequent irradiation (2 Gy, 1 h recovery), as indicated. The blots shown are representative of 3 independent experiments.

b, Illustration of the luciferase splicing reporter vectors containing (Luc-I) or lacking (Luc) an intron that are stably integrated in HeLa cells. HeLa Luc cells express luciferase constitutively, whereas HeLa Luc-I cells express luciferase only when splicing is functional and the intron in the coding RNA has been spliced out.

c, Splicing activity in HeLa Luc and Luc-I cells treated with the GapmeRs indicated for 48 h or pladienolide B (an inhibitor of splicing by targeting the SF3b1 subunit of the spliceosome) for 16 h. Splicing activity (means ±_SD, n=3) is expressed as the ratio between the Luc-I and Luc luciferase values following normalization to the corresponding control GapmeR or DMSO value, **p<0.01, ns (not significant) as determined by one-way ANOVA and two-sided Šidák's multiple comparisons test.

d, qPCR analysis of the expression of scaRNA2 and snU2 in HeLa Luc-I cells following their knockdown employing GapmeRs. The RNA levels were normalized to those of β-actin mRNA and are shown relative to the Control sample (means ± SD, n=3), ***p<0.001 as determined by one-way ANOVA and two-sided Dunnett's multiple comparisons test for scaRNA2 and *p<0.05 as determined by unpaired two-tailed t-test for snU2.
Source data are provided as a Source Data file.

Supplementary Fig. 5: Loss of scaRNA2 results in long-term impairment of pATM foci

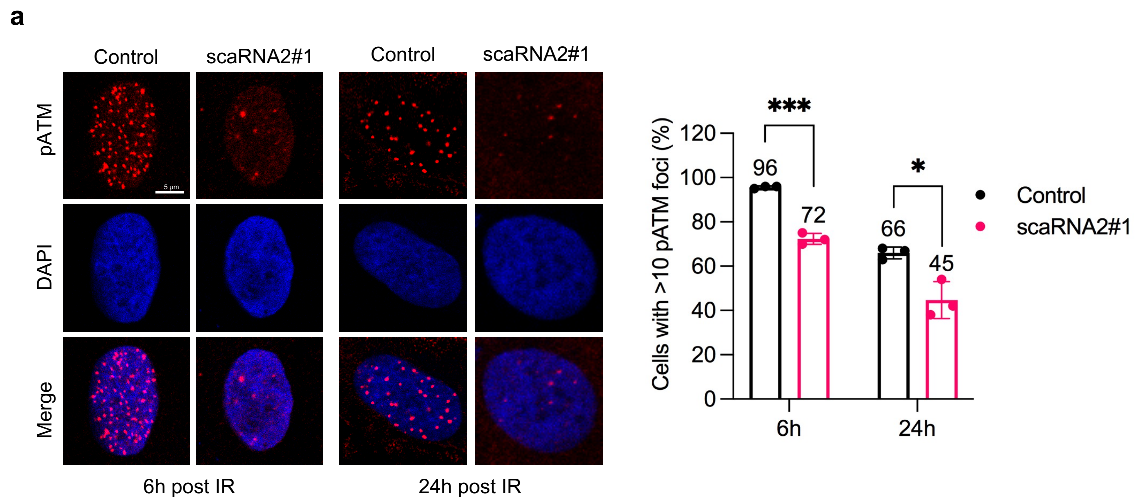

a, U2OS cells were transfected with GapmeRs for 48 h, irradiated (2 Gy), fixed after 1 h and immunostained for pATM. The graph illustrates the percentage of 100-200 cells (means ± SD, n=3) whose nuclei contained >10 IR-induced foci, *p<0.05, ***p<0.001 as determined by unpaired two-tailed t-test. Source data are provided as a Source Data file.

Supplementary Fig. 6: scaRNA2 binds to DNA-PK, but not to components of the MRN complex

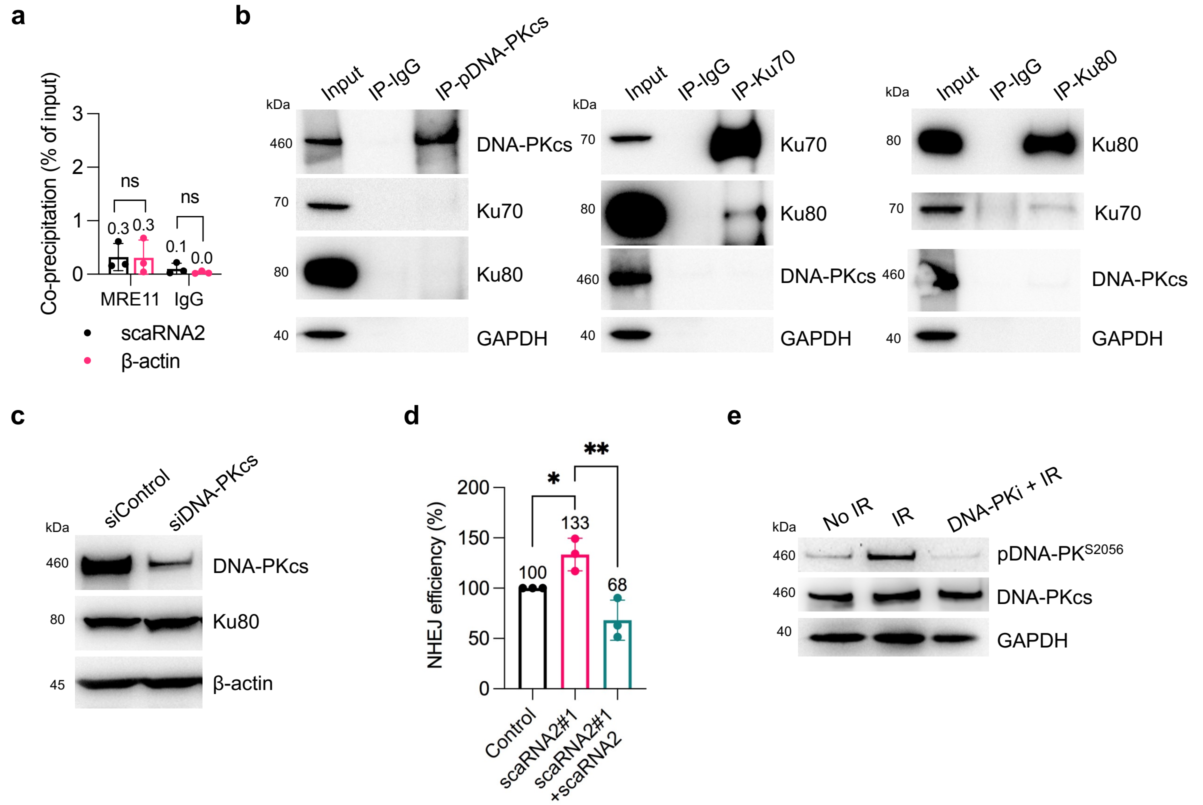

a, Native RIP of MRE11 or IgG from U2OS cells. The graphs show the amount of co-precipitated RNA as a percentage of input (means ± SD, n=3) measured by qPCR, ns (not significant) as determined by unpaired two-tailed t-test.

b, Representative blots of immunoprecipitation utilizing DNA-PKcs, Ku70, Ku80 or IgG antibodies in U2OS cells (n=3), also used for RIP in Fig. 4a.

c, Western blotting of indicated proteins in U2OS cells following knockdown of DNA-PKcs for 48 h. The blots shown are representative of 3 independent experiments.

d, The efficiency of NHEJ following transfection of U2OS EJ5-GFP NHEJ cells with the GapmeRs indicated for 8 h followed by transfection of a scaRNA2 plasmid for another 40 h. GFP expression was analyzed by flow cytometry. The values shown are relative to the Control sample (means ± SD, n=3), *p<0.05, **p<0.01 as determined by one-way ANOVA and two-sided Dunnett's multiple comparisons test.

e, U2OS cells were left untreated or treated with 4 µM DNA-PK inhibitor for 4 h, with or without subsequent irradiation (2 Gy) and their proteins extracted 30 min later. DNA-PK and GAPDH were then analyzed by western blotting. The blots shown are representative of 3 independent experiments.

Source data are provided as a Source Data file.

Supplementary Fig. 7: scaRNA2 inhibits DNA-PK by binding to DNA-PKcs and is released from this protein following irradiation

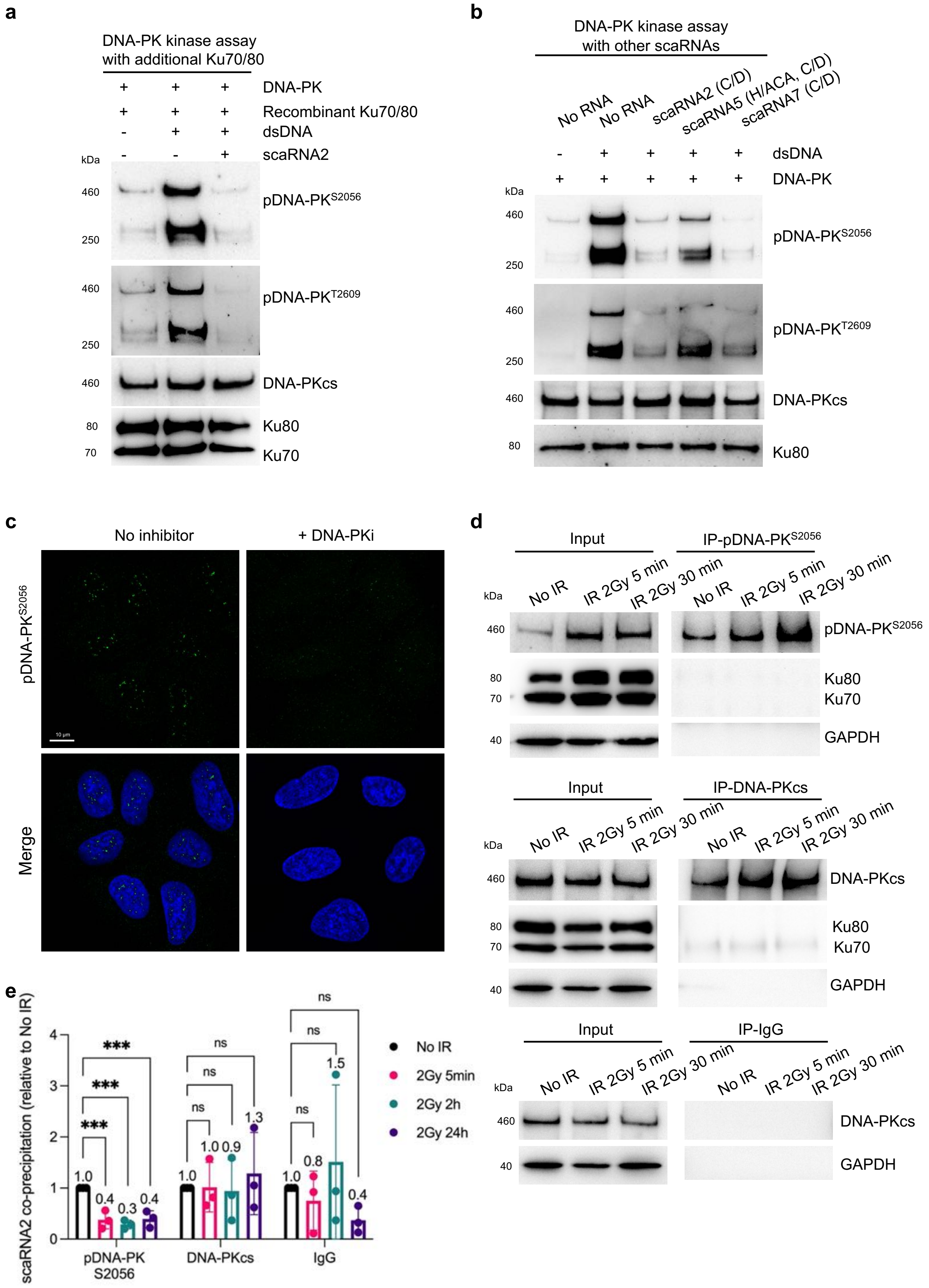

a, *In vitro* assay of DNA-PK kinase activity employing recombinant human DNA-PK (25-30 U), additional recombinant Ku70/80 (0.25 μg), double-stranded (ds) DNA (10 ng) or *in vitro* transcribed scaRNA2 (50 ng), followed by western blotting of the proteins indicated (n=3).

b, *In vitro* assay of DNA-PK kinase activity employing recombinant human DNA-PK (25-30 U), double-stranded (ds) DNA (10 ng) or *in vitro* transcribed scaRNA2, 5 or 7 (50 ng), followed by western blotting of the proteins indicated. The blots shown are representative of 3 independent experiments.

c, U2OS FokI cells were left untreated or treated with 10 μM DNA-PK inhibitor for 5 h, fixed and immunostained for pDNA-PK^{S2056} and DAPI (n=3).

d, Representative blots of immunoprecipitation utilizing DNA-PKcs, pDNA-PK^{S2056} or IgG antibodies in U2OS cells (n=3), also used for RIP in Fig. 5f.

e, Native RIP with pDNA-PK^{S2056}, total DNA-PKcs or IgG from U2OS cells that were irradiated (2 Gy) and left to recover for 5 min, 2 h or 24 h. The graph shows the amount of co-precipitated RNA relative to the non-irradiated sample (means ± SD, n=3) measured by qPCR, ***$p<0.001$, ns (not significant) as determined by one-way ANOVA and two-sided Dunnett's multiple comparisons test.

Source data are provided as a Source Data file.

Supplementary Fig. 8: DNA-PK assembly is enhanced following loss of scaRNA2 and the 18-2 antibody against DNA-PKcs cannot co-precipitate scaRNA2

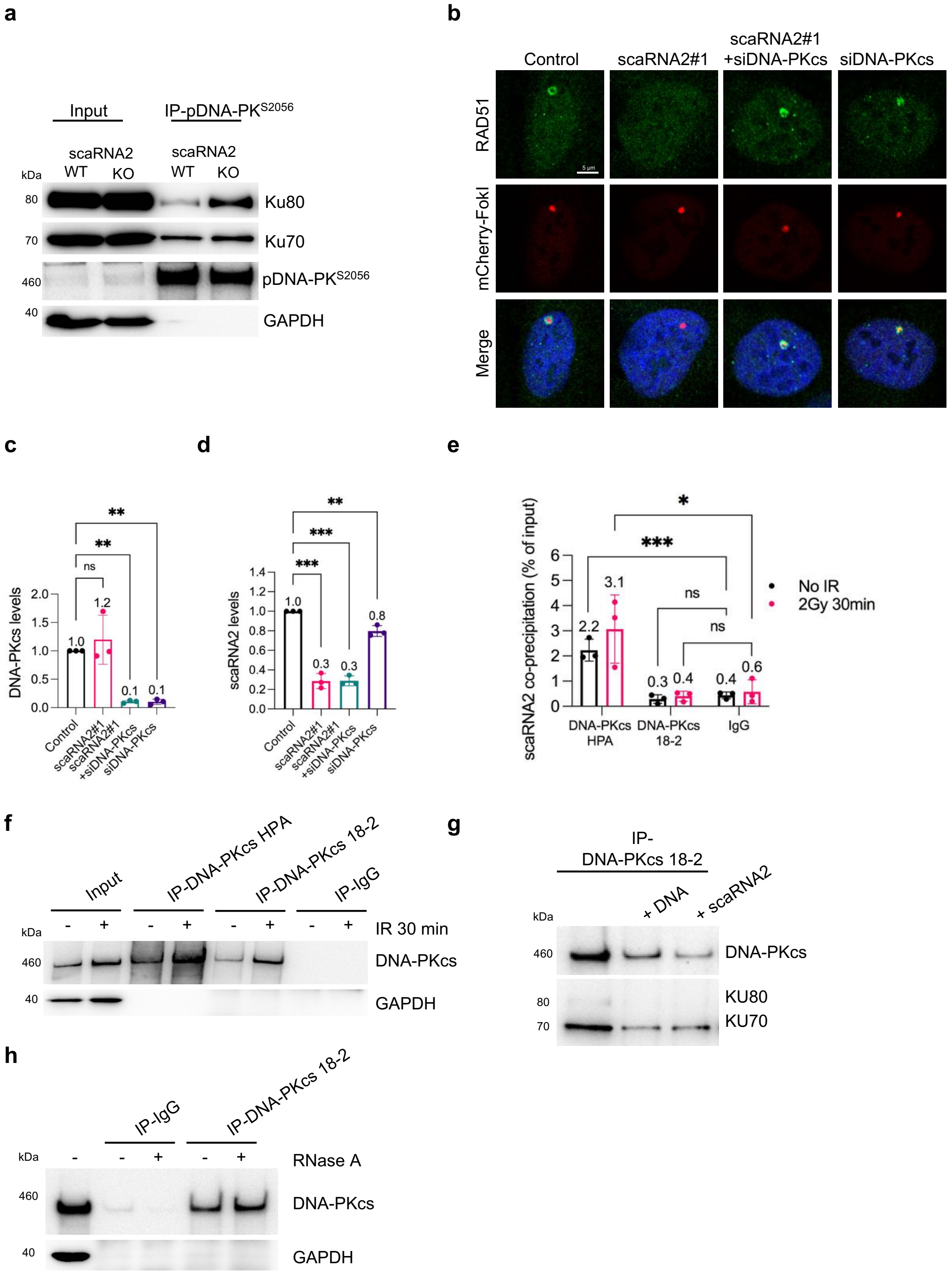

a, IP of pDNA-PK^{S2056} or IgG in irradiated (6 Gy, 30 min recovery) U2OS cells, stably depleted of scaRNA2 as indicated. Western blots show co-precipitated proteins (n=3).

b, U2OS FokI cells were transfected with GapmeRs or siRNAs directed against scaRNA2 or DNA-PKcs alone or in combination for 48 h, treated with Shield and 4-OHT for an additional 4 h, fixed and immunostained for RAD51 (n=3), related to Fig. 6c.

c, d, qPCR analysis of the expression of (c) DNA-PKcs and (d) scaRNA2 in U2OS FokI cells following their knockdown for 48 h. The RNA levels were normalized to those of β-actin mRNA and are shown relative to the Control sample (means ± SD, n=3), **p<0.01, ***p<0.001, ns (not significant) as determined by one-way ANOVA and two-sided Dunnett's multiple comparisons test.

e, RIP of DNA-PKcs from untreated or irradiated (2 Gy, 30 min recovery) U2OS cells employing two different antibodies or IgG. The graph shows the amount of co-precipitated scaRNA2 as a percentage of input (means ± SD, n=3) measured by qPCR, *p<0.05, ***p<0.001, ns (not significant) as determined by one-way ANOVA and two-sided Dunnett's multiple comparisons test.

f, Representative blots of immunoprecipitation utilizing the DNA-PKcs antibodies shown in Supplementary Fig. 8e (n=3).

g, IP of recombinant DNA-PK in the absence or presence of DNA (linearized plasmid) or RNA (*in vitro* transcribed scaRNA2) employing the 18-2 antibody. The blots shown are representative of 2 independent experiments.

h, IP of DNA-PKcs from untreated or RNase A-treated U2OS cells employing the 18-2 antibody or IgG. The blots show is representative of 2 independent experiments.

Source data are provided as a Source Data file.

Supplementary Fig. 9: Loss of LINP1 from cells lacking scaRNA2 restores both hyperphosphorylation of DNA-PK and the assembly of RAD51 at DNA breaks.

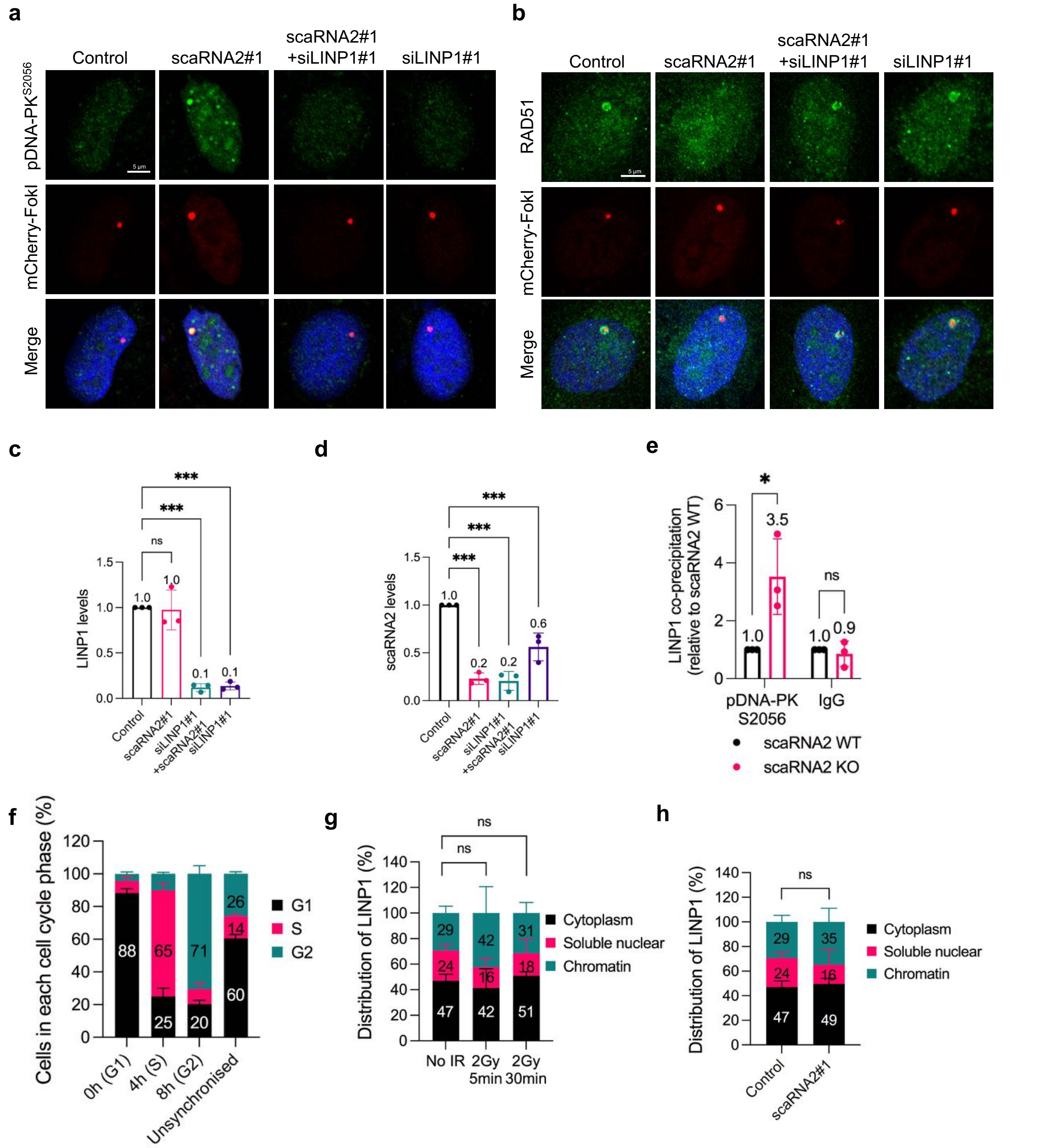

a, b, U2OS FokI cells were transfected with GapmeRs or siRNAs directed against scaRNA2 or LINP1 alone or in combination for 48 h, treated with Shield and 4-OHT for an additional 4 h, fixed and immunostained for (a) pDNA-PK^{S2056} or (b) RAD51, related to Fig. 6d, e.

c, d, qPCR analysis of the expression of (c) LINP1 and (d) scaRNA2 in U2OS FokI cells following their knockdown for 48 h. The RNA levels were normalized to those of β-actin mRNA and are shown relative to the Control sample (means ± SD, n=3), **$p<0.01$,

***p<0.001, ns (not significant) as determined by one-way ANOVA and two-sided Dunnett's multiple comparisons test.

e, RIP of pDNA-PK^{S2056} in irradiated (6 Gy, 30 min recovery) U2OS scaRNA2 WT or KO cells. The graph depicts the amount of RNA co-precipitated (means ± SD, n=3), as measured by qPCR and presented relative to the scaRNA WT sample, *p<0.05, ns (not significant) as determined by unpaired two-tailed t-test.

f, Flow cytometric characterization of the cell cycle of unsynchronized or synchronized U2OS cells. The percentages of cells (means ± SD, n=3) in the G1, S and G2 phases of the cell cycle are shown.

g, Distribution of LINP1 in the cytoplasmic, soluble nuclear and chromatin fractions of untreated or irradiated (2 Gy, 5 or 30 min recovery) U2OS cells as determined by qPCR. The values shown are means ± SD, n=3, ns (not significant) as determined by one-way ANOVA and two-sided Dunnett's multiple comparisons test.

h, Distribution of LINP1 in the cytoplasmic, soluble nuclear and chromatin fractions of U2OS cells treated with the GapmeRs indicated for 48 h, as determined by qPCR. The values shown are means ± SD, n=3, ns (not significant) as determined by unpaired two-tailed t-test.

Source data are provided as a Source Data file.

Supplementary Fig. 10: Loss of WRAP53β attenuates autophosphorylation of DNA-PK

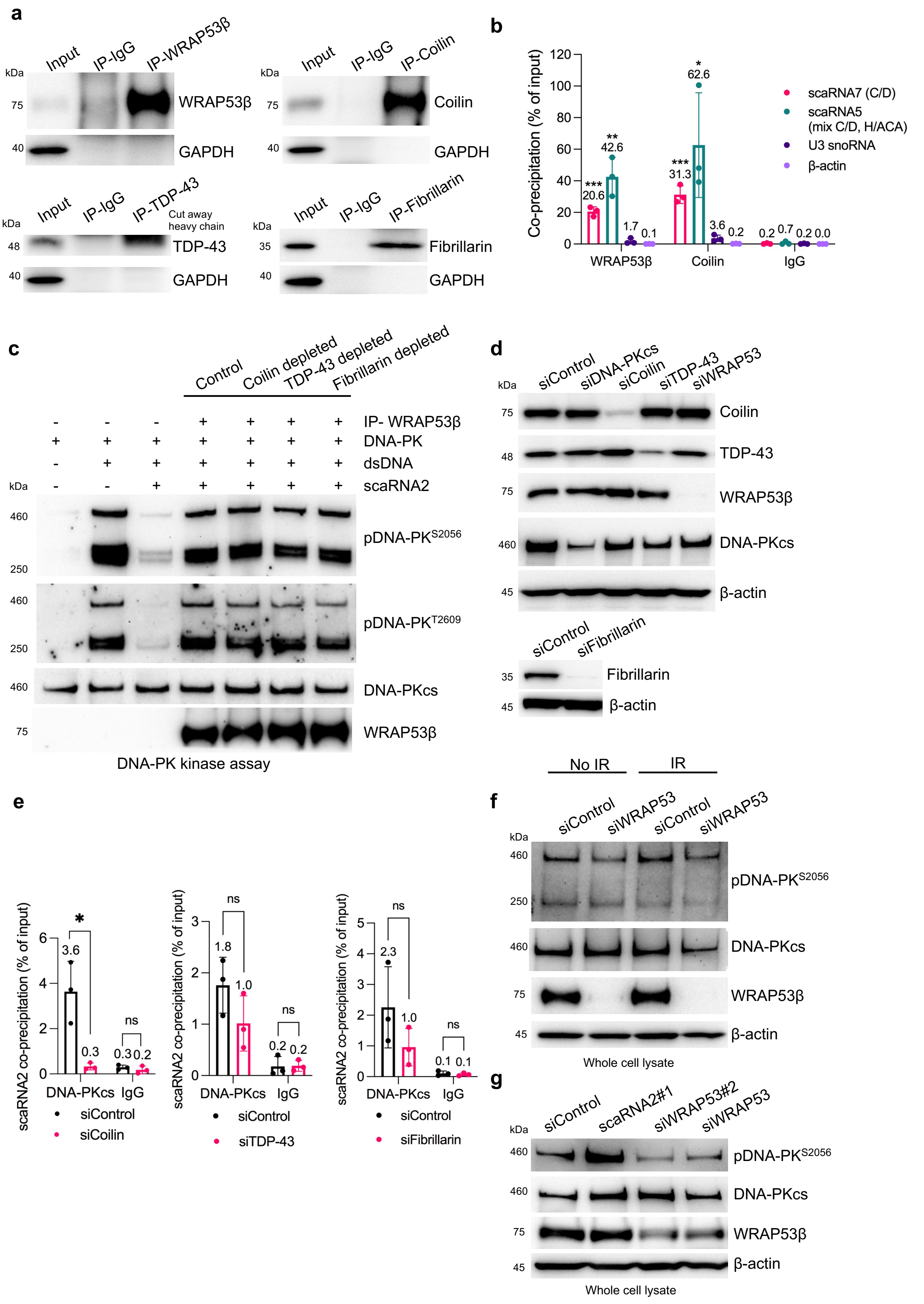

a, Representative blots of immunoprecipitation utilizing WRAP53β, coilin, TDP-43, fibrillarin or IgG antibodies in U2OS cells (n=3), used for CLIP in Fig. 7a and Supplementary Fig. 10b.

b, CLIP of WRAP53β, coilin, TDP-43, fibrillarin or IgG from U2OS cells. The graphs show the amount of co-precipitated RNA as a percentage of input (means ± SD, n=3) measured by qPCR, significant differences between the RNA of interest and β-actin are depicted, *p<0.05, ** *p*<0.01, ***p<0.001 as determined by unpaired two-tailed t-test. Representative blots depicting the immunoprecipitation of WRAP53β, coilin, TDP-43, fibrillarin or IgG are presented in Supplementary Fig. 10a.

c, *in vitro* assay of DNA-PK kinase activity employing recombinant human DNA-PK (25-30U), dsDNA (10 ng), scaRNA2 (100 ng) and/or WRAP53β protein obtained by IP from U2OS cells depleted of coilin, TDP-43 or fibrillarin for 48 h, followed by western blotting of the proteins indicated. The blots shown are representative of 3 independent experiments.

d, Western blotting of indicated proteins in U2OS cells following knockdown of DNA-PKcs, coilin, TDP-43, WRAP53β or fibrillarin for 48 h (n=3).

e, RIP of DNA-PKcs or IgG from U2OS cells transiently depleted of coilin, TDP-43 or fibrillarin as indicated. The graph shows the amount of co-precipitated RNA as a percentage of input (means ± SD, n=3) measured by qPCR, *p<0.05, ns (not significant) as determined by unpaired two-tailed t-test.

f, U2OS cells were depleted of WRAP53β for 48 h, with or without subsequent irradiation (2 Gy), and their proteins extracted 5 min later for western blotting. The blots shown are representative of > 3 independent experiments.

g, U2OS cells were transfected with GapmeRs or siRNA for 48 h, then irradiated (2 Gy) and 30 min later their proteins extracted for western blotting. The blots shown are representative of > 3 independent experiments.

Source data are provided as a Source Data file.

Supplementary Table 1. scaRNAs, their host genes and the functions of the host gene products. * scaRNAs coded by genes also encoding proteins involved in DNA repair and chromatin organization. scaRNA2 has been highlighted in yellow.

scaRNA gene	Host Gene	Function of host gene
	**Protein-encoding host gene**	
SCARNA1	*PPP1R8* (protein phosphatase 1 regulatory subunit 8)	Inhibitor of type 1 serine/threonine protein phosphatases involved in chromatin modification and transcription regulation.
SCARNA3 *	*COP1* (constitutive photomorphogenic 1 E3 ubiquitin ligase)	E3 ligase involved in the ubiquitin proteasome system. Ubiquitinates transcription factors, including p53 and MSH2 involved in DNA repair.
SCARNA4	*KHDC4* (K homology (KH) domain containing 4, pre-mRNA splicing factor)	RNA-binding protein involved in pre-mRNA splicing during transcription.
SCARNA5	*ATG16L1* (autophagy related 16 like 1)	Scaffold protein essential for autophagy and autophagosome formation. Also involved in the ubiquitin proteasome system.
SCARNA6	*ATG16L1* (autophagy related 16 like 1)	Same as above.
SCARNA7 *	*KPNA4* (karyopherin subunit alpha 4)	Nuclear import factor. Implicated in DNA repair through the nuclear import of repair factor XPA.
SCARNA8 *	*HAUS6* (HAUS augmin like complex subunit 6)	Part of the augmin complex involved in microtubule formation and organization important for chromosome segregation.
SCARNA9 *	*CEP295* (centrosomal protein 295)	Scaffold protein involved in centriolar assembly and microtubule association important for chromosome segregation.
SCARNA10 *	*NCAPD2* (non-SMC condensing I complex subunit D2	Part of the condensing I complex involved in mitotic chromosome condensation and segregation.
SCARNA11 *	*CHD4* (chromodomain helicase DNA binding protein 4)	Part of the nucleosome remodeling deacetylase (NuRD) complex involved in chromosome remodeling at sites of transcription and DNA repair.
SCARNA12 *	*PHB2* (prohibitin 2)	Mitochondrial membrane protein that mediates mitophagy. Also involved in sister-chromatid cohesion.
SCARNA14 *	*TIPIN* (TIMLESS interacting protein)	Part of the replisome and involved in DNA replication fork progression/protection, chromosome cohesion and DNA repair.
SCARNA16	*SEC14L1* (SEC14 like lipid binding 1)	Maybe involved in intracellular trafficking like other members of the SEC14 cytosolic factor family.
SCARNA18	*TMEM167A* (transmembrane protein 167A)	Transmembrane protein involved in vesicular trafficking.
SCARNA18B	*UCHL5* (ubiquitin C-terminal hydrolase L5)	Deubiquitinating enzyme involved in the ubiquitin proteasome system.
SCARNA20	*USP32* (ubiquitin specific peptidase 32)	Deubiquitinating enzyme involved in the ubiquitin proteasome system and vesicular trafficking.
SCARNA21 *	*CHD3* (chromodomain helicase DNA binding protein 3)	Part of the nucleosome remodeling deacetylase (NuRD) complex involved in chromosome remodeling at sites of transcription and DNA repair
SCARNA21B	*DNAJC16* (DnaJ heat shock protein family (Hsp40) member C16)	Member of the chaperone DnaJ complex that prevents misfolding of proteins.
SCARNA22 *	*NSD2* (nuclear receptor binding SET domain protein 2)	Histone methyltransferase involved in DNA repair and transcription regulation.
SCARNA23 *	*POLA1* (DNA polymerase alpha 1, catalytic subunit)	Catalytic subunit of the DNA polymerase alpha complex and part of the replisome. Involved in initiation of DNA replication.
SCARNA26A	*YY1AP1* (YY1 associated protein 1)	May collaborate with YY1 transcription factor.
SCARNA26B	*GON4L* (gon-4 like)	Chromatin factor involved in transcriptional regulation, some in collaboration with YY1 transcription factor.
SCARNA27 *	*EEF1E1* (eukaryotic translation elongation factor 1 epsilon)	Part of tRNA synthase complexes involved in protein translation. This protein also binds and stimulates ATM activity during DNA damage.
SCARNA28 *	*TRRAP* (transformation/transcription domain associated protein)	Member of the phosphatidylinositol 3-kinase related kinase (PIKK) family and part of several histone acetyltransferase (HAT) complexes involved in transcription and DNA repair.
	**Non-coding RNA host gene**	
SCARNA15	*SNHG21* (small nucleolar RNA host gene 21)	Encoded transcript is believed to be a vehicle for the production of scaRNA and snoRNA
SCARNA17 (U91)	*SNHG22* (small nucleolar RNA host gene 22)	Same as above.
SCARNA13 (U93)	*SNHG10* (small nucleolar RNA host gene 10)	Same as above.
	**No host gene**	
SCARNA2	Own promoter	
TERC (SCARNA19)	Own promoter	

Supplementary Table 2. The cells and culture conditions employed

Cell Line	Culture Conditions	Supplier
U2OS	DMEM	ATCC (HTB-96)
U2OS scaRNA2 WT or KO	DMEM	Generated within this study
MCF7-Cas9	DMEM plus 1 µg/ml puromycin	Gifted from Galina Selivanova (Karolinska, Institutet)
MCF7-Cas9 scaRNA2 WT or KO	DMEM plus 1 µg/ml puromycin	Generated within this study
p-Tuner 256 (U2OS FokI)	DMEM plus GlutaMAX	Gifted from Roger Greenberg (University of Pennsylvania)
HeLa Luc and Luc-I	DMEM plus 100 µg/ml Hygromycin B	Gifted from Gideon Dreyfuss (Howard Hughes Medical Institute)
U2OS DR-GFP HR reporter cells	DMEM plus 2 µg/ml puromycin	Gifted from Thomas Helleday (Karolinska Institutet)
U2OS EJ5-GFP NHEJ reporter cells	DMEM plus 2 µg/ml puromycin	Gifted from Jeremy Stark (Beckman Research Institute of the City of Hope)

Supplementary Table 3. The oligonucleotides and plasmids employed

	siRNAs and GapmeRs
siControl	Qiagen, catalog no. 1027280
siLINP1#1	Dharmacon, custom made. Product number: CTM-616412
siRAD51	Qiagen, catalog no. SI02663682
siWRAP53#2	Qiagen, catalog no. SI00388948
siWRAP53pool	Dharmacon, catalog no. M-021146-02-0005
siDNA-PKcs pool	Dharmacon, catalog no. L-005030-00-0005
siCoilin pool	Dharmacon, catalog no. L-019894-01-0005
siTDP-43 pool	Dharmacon, catalog no. L-012394-00-0005
siFibrillarin pool	Dharmacon, catalog no. M-011269-00-0005
GapmeR Control	AACACGTCTATACGC, Qiagen, custom made. Product number: 339516 LG00000002-DFA
GapmeR scaRNA2#1	GCTTCGCAGGAGGAGA, Qiagen, custom made. Design ID: 291108-1 Product number 339511 LG00085098-DDA
GapmeR scaRNA2#3	TCCGATCAAATAAGAT, Qiagen, custom made. Design ID: 291108-3 Product number: 339511 LG00085100-DDA
Gapmer snU2#5	Qiagen, custom made. Product number: 339512 LG00219338-DFA
	**Plasmids**
Flag-pCMV-Tag2B-empty	Agilent technologies #211172
pGEMT-easy scaRNA2	Gifted by Tamas Kiss (University of Toulouse, Toulouse)
I-SceI	Gifted by Thomas Helleday (Karolinska Institutet, Stockholm)
pMS2-GFP (GFP-MCP)	Addgene #27121
pX459 vector	Addgene #62988
pcDNA3_scaRNA2_deltaGU	Gifted by Michael Hebert (University of Mississippi Medical Center, USA)
pBS_scaRNA2_1-253	Gifted by Michael Hebert (University of Mississippi Medical Center, USA)
pBS_scaRNA2_254-420	Gifted by Michael Hebert (University of Mississippi Medical Center, USA)
miR-ScaRNA2	Mature scaRNA2 sequence cloned into a miR-146a plasmid (Plasmid #15092)
miR-ScaRNA5	Mature scaRNA5 sequence cloned into a miR-146a plasmid (Plasmid #15092)
miR-ScaRNA7	Mature scaRNA7 sequence cloned into a miR-146a plasmid (Plasmid #15092)
pGEMT-easy_scaRNA-MS2	MS2 sequence inserted at nucleotide 161
	**sgRNAs for CRISPR**
MCF7 Guide RNA #1 5'UTR	CGGAGAGCTTAAAAGTCGCG
MCF7 Guide RNA #2 3'UTR	AGGGGCGAGCACGTGAACGG
U2OS Guide RNA #1 5'UTR	GGGACCACAGGCGATGGCCA
U2OS Guide RNA #2 3'UTR	GCTGCCCCTCCCCCTGGAGC
	**Genotyping primers**
scaRNA2 Forward Primer	TGGGACCTATGTTCCCCTCC
scaRNA2 Reverse Primer	CAGCGGCTCATTTACCCTTG
	**qPCR Primers**
β-actin Forward Primer	AGGTCATCACCATTGGCAATGAG
β-actin Reverse Primer	CTTTGCGGATGTCCACGTCA

GAPDH Forward Primer	GCTTGCCCTGTCCAGTTAAT
GAPDH Reverse Primer	TAGCTCAGCTGCACCCTTTA
LINP1 Forward primer	AGCCGGTCCAGTACACCTTT
LINP1 Reverse primer	GGAAAGCACCGTCTGTTGTT
scaRNA2 Forward Primer	GCTTGGAGCGTGTTAGGC
scaRNA2 Reverse Primer	GGAGGAGAGCTTTTCATTTCG
snU2 Forward primer	GGCCTTTTGGCTAAGATCAAG
snU2 Reverse primer	GGAGCAAGCTCCTATTCCATC
DNA-PKcs Forward primer	GATCGACCTGAAGCGCC
DNA-PKcs Reverse primer	CTATTGGATTTCCCAGTTTGCCAT
MALAT1 Forward primer	AGTTCCGGGGGTTTTGTGAG
MALAT1 Reverse primer	AGCGTGTGGAAAGATTTGAGC
EEF2 Forward primer	AAACTGGACAGCGAGGACAA
EEF2 Reverse primer	TGGGGTCACAGCTTTTAATGC
scaRNA5 Forward primer	TGAATGTCACGGTCCCTTTGTTGC
scaRNA5 Reverse primer	AGCTGCTCCATGATCCCATACACA
scaRNA7 Forward primer	ACTGACTGGTGATGCTGATATT
scaRNA7 Reverse primer	GCTAGTTAGGTGAGCTGTTCTT
U3 snoRNA Forward primer	CGTGTAGAGCACCGAAAACC
U3 snoRNA Reverse primer	CGCTCAGGAGAAAACGCTAC
	**RNA FISH probes**
scaRNA2_1:	**AGTAGTTCAGACGCCGTTAA**AGGACCCAGGCCGCTCTCCCTCCCTAAAACA**AGTAGTTCAGACGCCGTTAA**
scaRNA2_2:	**AGTAGTTCAGACGCCGTTAA**GCGCCTCACACGCGACGCCACAGCTCCGCA**AGTAGTTCAGACGCCGTTAA**
scaRNA2_3:	**AGTAGTTCAGACGCCGTTAA**CACTCACACACTCACGCGTCCACTCACACT**AGTAGTTCAGACGCCGTTAA**
scaRNA2_4:	**AGTAGTTCAGACGCCGTTAA**GGTGGGCGCACGCACTCGCCTAACACGCTC**AGTAGTTCAGACGCCGTTAA**
scaRNA2_5:	**AGTAGTTCAGACGCCGTTAA**TCAAATAAGATCAAAGTGTAAGCGGGAGGA**AGTAGTTCAGACGCCGTTAA**
scaRNA2_6:	**AGTAGTTCAGACGCCGTTAA**CATTTCGGGTCGGCCCGGCGGGGCTGGGGT**AGTAGTTCAGACGCCGTTAA**
scaRNA2_7:	**AGTAGTTCAGACGCCGTTAA**GCACAGCGCCCCGAGGGGGCTTCGCAGGAG**AGTAGTTCAGACGCCGTTAA**
scaRNA2_8:	**AGTAGTTCAGACGCCGTTAA**TCGGGGACCACAGCGTGGCCGCCGCCTAAG**AGTAGTTCAGACGCCGTTAA**
scaRNA2_9:	**AGTAGTTCAGACGCCGTTAA**ACTTCTGAGCGCCGGGCCCCGCAGGGCCAG**AGTAGTTCAGACGCCGTTAA**
scaRNA2_10:	**AGTAGTTCAGACGCCGTTAA**CCGGGGACAAGCCCGGCCTCGTCTATCTGA**AGTAGTTCAGACGCCGTTAA**
U2 snRNA_1	**AGTAGTTCAGACGCCGTTAA**CACTTGATCTTAGCCAAAAGGCCGAGAAGC**AGTAGTTCAGACGCCGTTAA**
U2 snRNA_2	**AGTAGTTCAGACGCCGTTAA**CGTTCCTGGAGGTACTGCAATACCAGGTCGA**AGTAGTTCAGACGCCGTTAA**
Fluorescent Q1:	/5Alex488N/TTAACGGCGTCTGAACTACT/3Alex488N/
	**Mutagenesis Primers and Cloning Primers**
scaRNA2 1x**MS2** ins nt161 Forward primer	CCCCTGCGCCCCT**CGTACACCATCAGGGTACG**CCTCCCGCTTA
scaRNA2 1x**MS2** ins nt161 Reverse primer	TAAGCGGGAGG**CGTACCCTGATGGTGTACG**AGGGGCGCAGGGG
scaRNA2 cloning into MiR Forward primer	CTGCAGGGATCCGTTTTAGGGAGGGAGAGCGGCCTGGGT
scaRNA2 cloning into MiR Reverse primer	GAATTCCTCGAGCCAGATCAGAATCGCCTCGATAAT

scaRNA5 cloning into MiR Forward primer	CTGCAGGGATCCAGGTCGATGATGATTGGTAAAAGGTCT
scaRNA5 cloning into MiR Reverse primer	GAATTCCTCGAGAGGTCTCAGATTGAAAACTTGAGA
scaRNA7 cloning into MiR Forward primer	CTGCAGGGATCCTGGAAATGATGAAATAGAGATAATTGG
scaRNA7 cloning into MiR Reverse primer	GAATTCCTCGAGTTGGATCAGATCACAGGACCATTT
LINP1-T7 Forward primer	TAATACGACTCACTATAGGGCCCTCCGTCTCCTTGACTCT
LINP1 Reverse primer	TTTTTTTTATTTCTAAATCCTTTAGTCATTTGGC

Supplementary Table 4. The antibodies employed

Antibodies	Catalogue No	Supplier	Species	Used for	Dilution IF	Dilution WB
53BP1	NB100-904	Novus biologica	Rabbit	IF, WB	1/200	1/1000
ATM	sc-23921	Santa Cruz	Mouse	IF, WB	1/100	1/1000
ß-actin	A5316	Sigma-Aldrich	Mouse	WB	-	1/10.000
BRCA1	sc-6954	Santa Cruz	Mouse	IF, WB	1/50	1/250
Coilin	sc-56298	Santa Cruz	Mouse	IF, IP	1/50	-
Coilin	ab210785	Abcam	Rabbit	IP	-	-
CTCF	ab70303	Abcam	Rabbit	WB	-	1/1000
CtIP	61142	Active Motif	Mouse	IF, WB	1/50	1/1000
DNA ligase IV	GTX55592	Genetex	Rabbit	IF	1/50	-
DNA-PKcs	HPA035174	Atlas	Rabbit	IP, WB	-	1/1000
DNA-PKcs	A303-967A	Bethyl	Goat	IP	-	-
DNA-PKcs	MA5-13238	Invitrogen	Mouse	IF, IP, WB	1/100	1/1000
Fibrillarin	ab5821	Abcam	Rabbit	IP, WB	-	1/1000
FK2	BML-PW8810	Enzo	Mouse	IF	1/50	-
GAPDH	sc47724	Santa Cruz	Mouse	WB	-	1/2000
H2AX	ab11175	Abcam	Rabbit	WB	-	1/2000
H2B	ab1790	Abcam	Rabbit	WB	-	1/1000
HSP90	sc-13119	Santa Cruz	Mouse	WB	-	1/1000
IgG	12-371	Millipore	Mouse	IP	-	-
IgG	12-370	Millipore	Rabbit	IP	-	-
Ku70	ab83501	Abcam	Rabbit	IF	1/100	-
Ku70	MA5-32645	Invitrogen	Rabbit	IP, WB	-	1/1000
Ku80	MA5-12933	Invitrogen	Mouse	IF, IP, WB	1/100	1/1000
MRE11	NB-100-142	Novus	Rabbit	IF, IP, WB	1/50	1/1000
NBS1	NB100-143	Novus	Rabbit	IF, WB	1/50	1/1000
pATM S1981	200-301-400	Rockland	Mouse	IF, WB	1/50	1/1000
pDNA-PK S2056	ab18192	Abcam	Rabbit	IP, WB	-	1/1000
pDNA-PK S2056	ab124918	Abcam	Rabbit	IF	1/50	-
pDNA-PK T2609	pA1-29541	Invitrogen	Rabbit	IF, WB	1/50	1/1000
RAD50	GTX70228	Genetex	Mouse	IF, WB	1/50	1/1000
RAD51	ab63801	Abcam	Rabbit	IF, WB	1/50	1/1000
RAD51	ABE257	Millipore	Rabbit	IF	1/50	-
RNF8	sc-271462	Santa Cruz	Mouse	WB	-	1/1000
RPA32/RPA2	ab2175	Abcam	Mouse	IF, WB	1/50	1/1000
TDP-43	ab57105	Abcam	Mouse	IP, WB	-	1/1000
WRAP53 (1F12)	H00055135-MO4	Abnova	Mouse	IF	1/50	-
WRAP53 (535)	PA-2020-100	Innovagen	Rabbit	WB, IP	-	1/1000
XRCC4	HPA006801	Atlas	Rabbit	IF	1/50	-
γH2AX	05-636	Millipore	Mouse	IF, WB	1/200	1/500
γH2AX	2577	Cell Signalling	Rabbit	IF	1/50	-
**Secondary Antibody**	**Catalogue No**	**Supplier**	**Species**	**Used for**		
Goat anti- mouse IgG, Alexa Fluor 488	A-11029	Thermo Fisher	Mouse	IF	1/1000	-
Goat anti- rabbit IgG, Alexa Fluor 488	A-11008	Thermo Fisher	Rabbit	IF	1/1000	-
Donkey anti- mouse IgG, Alexa Fluor 594	A-21203	Thermo Fisher	Mouse	IF	1/1000	-
Donkey anti- rabbit IgG, Alexa Fluor 594	A-21207	Thermo Fisher	Rabbit	IF	1/1000	-
A-mouse IgG, HRP linked antibody	7076S	Cell Signaling	Mouse	WB	-	1/10.000

A-rabbit IgG, HRP linked antibody	7074S	Cell Signaling	Rabbit	WB	-	1/10.000
