# Supplementary material for: Small Cajal body-associated RNA 2 (scaRNA2) regulates DNA repair pathway choice by inhibiting DNA-PK
Source: Nat Commun. 2022 Feb 23;13:1015. doi: 10.1038/s41467-022-28646-5 (PMC8866460; doi:10.1038/s41467-022-28646-5)
Supplement: Supplementary file 3 — Reporting Summary [file 41467_2022_28646_MOESM3_ESM.pdf]

nature portfolio

Corresponding author(s): Marianne Farnebo

Last updated by author(s): December 20, 2021

Reporting Summary

Nature Portfolio wishes to improve the reproducibility of the work that we publish. This form provides structure for consistency and transparency in reporting. For further information on Nature Portfolio policies, see our Editorial Policies and the Editorial Policy Checklist.

Statistics

For all statistical analyses, confirm that the following items are present in the figure legend, table legend, main text, or Methods section.

n/a	Confirmed	
☐	☒	The exact sample size (*n*) for each experimental group/condition, given as a discrete number and unit of measurement
☐	☒	A statement on whether measurements were taken from distinct samples or whether the same sample was measured repeatedly
☐	☒	The statistical test(s) used AND whether they are one- or two-sided<br>*Only common tests should be described solely by name; describe more complex techniques in the Methods section.*
☒	☐	A description of all covariates tested
☒	☐	A description of any assumptions or corrections, such as tests of normality and adjustment for multiple comparisons
☐	☒	A full description of the statistical parameters including central tendency (e.g. means) or other basic estimates (e.g. regression coefficient) AND variation (e.g. standard deviation) or associated estimates of uncertainty (e.g. confidence intervals)
☐	☒	For null hypothesis testing, the test statistic (e.g. *F*, *t*, *r*) with confidence intervals, effect sizes, degrees of freedom and *P* value noted<br>*Give P values as exact values whenever suitable.*
☒	☐	For Bayesian analysis, information on the choice of priors and Markov chain Monte Carlo settings
☒	☐	For hierarchical and complex designs, identification of the appropriate level for tests and full reporting of outcomes
☒	☐	Estimates of effect sizes (e.g. Cohen's *d*, Pearson's *r*), indicating how they were calculated

Our web collection on statistics for biologists contains articles on many of the points above.

Software and code

Policy information about availability of computer code

Data collection	No software were used
Data analysis	Prism GraphPad software v9.2.0, BD FACSDiva v8.0.2 (BD Biosciences), FlowJo v9 (BD Biosciences), Zen 2012 Black software (Zeiss), ImageJ (Java 1.8.0_172 64-bit), CellProfiler (version 3.1.8)

For manuscripts utilizing custom algorithms or software that are central to the research but not yet described in published literature, software must be made available to editors and reviewers. We strongly encourage code deposition in a community repository (e.g. GitHub). See the Nature Portfolio guidelines for submitting code & software for further information.

Data

Policy information about availability of data

All manuscripts must include a data availability statement. This statement should provide the following information, where applicable:

- Accession codes, unique identifiers, or web links for publicly available datasets
- A description of any restrictions on data availability
- For clinical datasets or third party data, please ensure that the statement adheres to our policy

Source data are provided with this paper. All other data supporting the findings of this study are available from the corresponding author on reasonable request.

Field-specific reporting

Please select the one below that is the best fit for your research. If you are not sure, read the appropriate sections before making your selection.

☒ Life sciences ☐ Behavioural & social sciences ☐ Ecological, evolutionary & environmental sciences

For a reference copy of the document with all sections, see nature.com/documents/nr-reporting-summary-flat.pdf

Life sciences study design

All studies must disclose on these points even when the disclosure is negative.

Sample size	No statistical method was used to predetermine sample size, but we routinely employed at least three biological repeats for each experiment. In the case of scoring cells, typically 50-200 cells were scored each time.
Data exclusions	Data exclusion was not applied
Replication	All experiments were independently replicated at least 3 times, and even more in case the experiment failed due to technical reasons
Randomization	Randomization was not applied as these experiments were performed on the same cell lines
Blinding	Blinding was not performed as this is not a standard procedure for these types of experiments, however, our reported findings were independently observed by more than one person

Reporting for specific materials, systems and methods

We require information from authors about some types of materials, experimental systems and methods used in many studies. Here, indicate whether each material, system or method listed is relevant to your study. If you are not sure if a list item applies to your research, read the appropriate section before selecting a response.

Materials & experimental systems

n/a	Involved in the study	
☐	☒	Antibodies
☐	☒	Eukaryotic cell lines
☒	☐	Palaeontology and archaeology
☒	☐	Animals and other organisms
☒	☐	Human research participants
☒	☐	Clinical data
☒	☐	Dual use research of concern

Methods

n/a	Involved in the study	
☒	☐	ChIP-seq
☐	☒	Flow cytometry
☒	☐	MRI-based neuroimaging

Antibodies

Antibodies used

Antibodies employed are also listed in Supplementary table 4
53BP1 (NB100-904 Novus biologica) IF: 1/200, WB: 1/1000
ATM (sc-23921 Santa Cruz) IF: 1/100, WB: 1/1000
ß-actin (A5316 Sigma-Aldrich) WB: 1/10000
BRCA1 (sc-6954 Santa Cruz) IF: 1/50, WB: 1/250
Coilin (sc-56298 Santa Cruz) IF: 1/50
Coilin (ab210785 Abcam)
CTCF (ab70303 Abcam) WB: 1/1000
CtIP (61142 Active Motif) IF: 1/50, WB: 1/1000
DNA ligase IV (GTX55592 Genetex) IF: 1/50
DNA-PKcs (HPA035174 Atlas) WB: 1/1000
DNA-PKcs (A303-967A Bethyl)
DNA-PKcs (MA5-13238 Invitrogen) IF: 1/100 WB: 1/1000
Fibrillarin (ab5821 Abcam) WB: 1/1000
FK2 (BML-PW8810 Enzo) IF: 1/50
GAPDH (sc47724 Santa Cruz) WB: 1/2000
H2AX (ab11175 Abcam) WB: 1/2000
H2B (ab1790 Abcam) WB: 1/1000
HSP90 (sc-13119 Santa Cruz) WB: 1/1000
IgG (12-371 Millipore)
IgG (12-370 Millipore)
Ku70 (ab83501 Abcam) IF: 1/100
Ku70 (MA5-32645 Invitrogen) WB: 1/1000

Ku80 (MA5-12933 Invitrogen) IF: 1/100, WB: 1/1000
MRE11 (NB-100-142 Novus) IF: 1/50, WB: 1/1000
NBS1 (NB100-143 Novus) IF: 1/50, WB: 1/1000
pATM S1981 (200-301-400 Rockland) IF: 1/50, WB: 1/1000
pDNA-PK S2056 (ab18192 Abcam) WB: 1/1000
pDNA-PK S2056 (ab124918 Abcam) IF: 1/50
pDNA-PK T2609 (pA1-29541 Invitrogen) IF: 1/50, WB: 1/1000
RAD50 (GTX70228 Genetex) IF: 1/50, WB: 1/1000
RAD51 (ab63801 Abcam) IF: 1/50, WB: 1/1000
RAD51 (ABE257 Millipore) IF: 1/50
RNF8 (sc-271462 Santa Cruz) WB: 1/1000
RPA32/RPA2 (ab2175 Abcam) IF: 1/50, WB: 1/1000
TDP-43 (ab57105 Abcam) WB: 1/1000
WRAP53 (1F12) (H00055135-MO4 Abnova) IF: 1/50
WRAP53 (535) (PA-2020-100 Innovagen) WB: 1/1000
XRCC4 (HPA006801 Atlas) IF: 1/50
γH2AX (05-636 Millipore) IF: 1/200 WB: 1/500
γH2AX (2577 Cell Signalling) IF: 1/50
Goat anti- mouse IgG, Alexa Fluor 488 (A-11029 Thermo Fisher) IF: 1/1000
Goat anti- rabbit IgG, Alexa Fluor 488 (A-11008 Thermo Fisher) IF: 1/1000
Donkey anti- mouse IgG, Alexa Fluor 594 (A-21203 Thermo Fisher) IF: 1/1000
Donkey anti- rabbit IgG, Alexa Fluor 594 (A-21207 Thermo Fisher) IF: 1/1000
A-mouse IgG, HRP linked antibody (7076S Cell Signaling) WB: 1/10000
A-rabbit IgG, HRP linked antibody (7074S Cell Signaling) WB: 1/10000

If no dilutions are given the antibody was only used for IP

Validation

The specificity of all antibodies employed were confirmed either by us, published litterature or the company that sold them. Internal validation by us included the use of cell lines lacking the target antigen.

Eukaryotic cell lines

Policy information about cell lines

Cell line source(s)

U2OS were purchased from ATCC, U2OS scaRNA2 WT/KO and MCF7-Cas9 scaRNA2 WT/KO were generated within this study, MCF7-Cas9 were gifted from Galina Selivanova (Karolinska, Institutet), p-Tuner 256 (U2OS FokI) were gifted from Roger Greenberg (University of Pennsylvania), HeLa Luc and Luc-I were gifted from Gideon Dreyfuss (Howard Hughes Medical Institute), U2OS DR-GFP HR reporter cells were gifted from Thomas Helleday (Karolinska Institutet), U2OS EJ5-GFP NHEJ reporter cells were gifted from Jeremy Stark (Beckman Research Institute of the City of Hope). Also listed in Supplementary Table 2

Authentication

PCR authentication performed

Mycoplasma contamination

All cell lines tested negative for mycoplasma

Commonly misidentified lines
(See ICLAC register)

The cell lines used in this study are not commonly misidentified

Flow Cytometry

Plots

Confirm that:

☒ The axis labels state the marker and fluorochrome used (e.g. CD4-FITC).

☒ The axis scales are clearly visible. Include numbers along axes only for bottom left plot of group (a 'group' is an analysis of identical markers).

☒ All plots are contour plots with outliers or pseudocolor plots.

☒ A numerical value for number of cells or percentage (with statistics) is provided.

Methodology

Sample preparation

Cells were fixed in ethanol and staind with propidium iodide for cell cycle analysis and fixed in PFA for GFP reporter analysis.

Instrument

BD LSR II (BD Biosciences)

Software

BD FACSDiva software v8.0.2 (BD Biosciences), with quantification carried out utilizing FlowJo v9 (BD Biosciences)

Cell population abundance

No sorting was performed

Gating strategy

For GFP reporter cells: The cells were gated based on FSC and SSC. Single cells were gated based on FSC and FSC-H. GFP-positive cells were gated in the appropriate channel.

For PI staining: The cells were gated based on FSC and SSC. Single cells were gated based on FSC and FSC-H. Cell cycle phases were gated in the appropriate channel.

☒ Tick this box to confirm that a figure exemplifying the gating strategy is provided in the Supplementary Information.
